# Unraveling the molecular interactions between α7 nicotinic receptor and a RIC3 variant associated with backward speech

**DOI:** 10.1007/s00018-024-05149-8

**Published:** 2024-03-12

**Authors:** Aditi Pradhan, Hayley Mounford, Jessica Peixinho, Edward Rea, Emmanouela Epeslidou, Julia S. Scott, Joanna Cull, Susan Maxwell, Richard Webster, David Beeson, Yin Yao Dong, Stefan Prekovic, Isabel Bermudez, Dianne F. Newbury

**Affiliations:** 1 Department of Biological and Molecular Sciences, Faculty of Health and Life Sciences, Oxford Brookes University, Oxford, OX3 0BP England; 2 Oxford Brookes Centre for Bioimaging, Oxford Brookes University, Oxford, OX3 0BP England; 3 Center for Molecular Medicine, University Medical Center Utrecht, Utrecht, The Netherlands; 4 Neurosciences Group, Weatherall Institute of Molecular Medicine, John Radcliffe Hospital, Oxford, OX3 9DS England; 5 Nuffield Department of Clinical Neurosciences, University of Oxford, Oxford, OX3 9DS England

**Keywords:** RIC3, Nicotinic acetylcholine receptors, Backward speech

## Abstract

Recent work putatively linked a rare genetic variant of the chaperone Resistant to Inhibitors of acetylcholinesterase (*RIC3*) (NM_024557.4:c.262G > A, NP_078833.3:p.G88R) to a unique ability to speak backwards, a language skill that is associated with exceptional working memory capacity. RIC3 is important for the folding, maturation, and functional expression of α7 nicotinic acetylcholine receptors (nAChR). We compared and contrasted the effects of RIC3G88R on assembly, cell surface expression, and function of human α7 receptors using fluorescent protein tagged α7 nAChR and Förster resonance energy transfer (FRET) microscopy imaging in combination with functional assays and ^125^I-α-bungarotoxin binding. As expected, the wild-type RIC3 protein was found to increase both cell surface and functional expression of α7 receptors. In contrast, the variant form of RIC3 decreased both. FRET analysis showed that RICG88R increased the interactions between RIC3 and α7 protein in the endoplasmic reticulum. These results provide interesting and novel data to show that a RIC3 variant alters the interaction of RIC3 and α7, which translates to decreased cell surface and functional expression of α7 nAChR.

**Supplementary Information:**

The online version contains supplementary material available at 10.1007/s00018-024-05149-8.

## Introduction

The homomeric α7 nicotinic acetylcholine receptor (nAChR) is one of the most abundant nAChRs in the brain. It is highly expressed in the hippocampus, thalamus, and cortex and contributes to cognition, attention, and working memory [1–3]. Our understanding of the exact links between α7 nAChR and cognitive functions are limited but validated links exist between α7 nAChRs and cognitive deficits associated with schizophrenia [3–5] and with Alzheimer’s disease, in which α7 is proposed to exert a neuroprotective effect [6, 7]. Furthermore, single nucleotide polymorphisms (SNPs) in the *CHRNA7* gene (which encodes the α7 subunit) have been associated with dementia [8], Alzheimer's disease [9, 10] and schizophrenia [11]. Although α7 usually forms homopentamers, in basal forebrain neurones, it can also assemble with β2 nAChR subunits to form heteromeric α7β2 nAChRs, thus increasing its functional range [12]. The diversity of α7 signaling is further enhanced by the ability of α7 nAChR to link with G-proteins, diverse intracellular signal pathways, and modulate intracellular calcium release from the endoplasmic reticulum (ER) [13].

To exert its signaling functions, α7 nAChR must be present on the cell surface, which largely depends on the correct folding and assembly of the receptor subunits in the ER and subsequent trafficking of the assembled receptor to the cell surface [14]. Robust experimental evidence indicates that the ER-resident chaperone RIC3 enhances α7 subunit folding and oligomerization in the ER leading to “mature” assemblies that are then trafficked to the cell surface [15–18]. In host cells that do not express RIC3, heterologous expression of the chaperone cDNA enables functional expression of α7 nAChR [15, 19, 20]. RIC3 also interacts with other nAChRs and the closely related 5-HT3 serotonin receptor but its effects, which can be positive or negative, depend on the identity of the receptor subunits, the host cell [16, 19] and the ratio of receptor to RIC3 [21, 22]. Although the role of RIC3 on the expression of α7 nAChR in vivo is not fully understood [23], *RIC3* expression has been linked to cognitive maintenance [24] and, crucially, there is a good correspondence between *RIC3* and α7 nAChR expression in the rat hippocampus [25]. Furthermore, recent autoradiographic analysis of the brain of a *Ric3* knockout mouse show a decrease in ^125^I-α-bungarotoxin binding in the hippocampus and the cortex [23], brain regions that contribute to working memory and language. In addition, the expression of *RIC3* shows a high level of correlation with α7 nAChR in postmortem brain tissues from population and disease cohorts [26].

Our interest in RIC3 stems from its potential role in language. Several studies have linked copy number changes of chromosome 15q13.3 (the location of the *CHRNA7* gene) with an increased risk of speech and language disorders, usually alongside more global developmental delays and neuropsychiatric phenotypes [27–31]. Although these chromosome rearrangements typically include 1.5–2 Mb of DNA and seven genes, smaller deletions affecting only *CHRNA7* result in similar developmental profiles, leading some to suggest that haploinsufficiency of *CHRNA7* underlies some of the features seen in this syndrome [32–34]. *RIC3* expression is specifically upregulated in both patients with schizophrenia and bipolar disorder [26], both of which include language dysfunction [35–37]. Furthermore, a recent study investigated a case family with the unique ability to speak backwards, a language skill that they postulated was made possible by exceptional working memory capacity [38]. This study identified three potential contributory variants including a rare polymorphism in *RIC3* which confers a coding change (NM_024557.4:c.262G > A, NP_078833.3:p.G88R) [38]. The genetic and behavioral bases for this skill remain unknown but the putative implication of RIC3 provides an intriguing link that we aim to substantiate on a functional level in this paper.

**Fig. 1:**
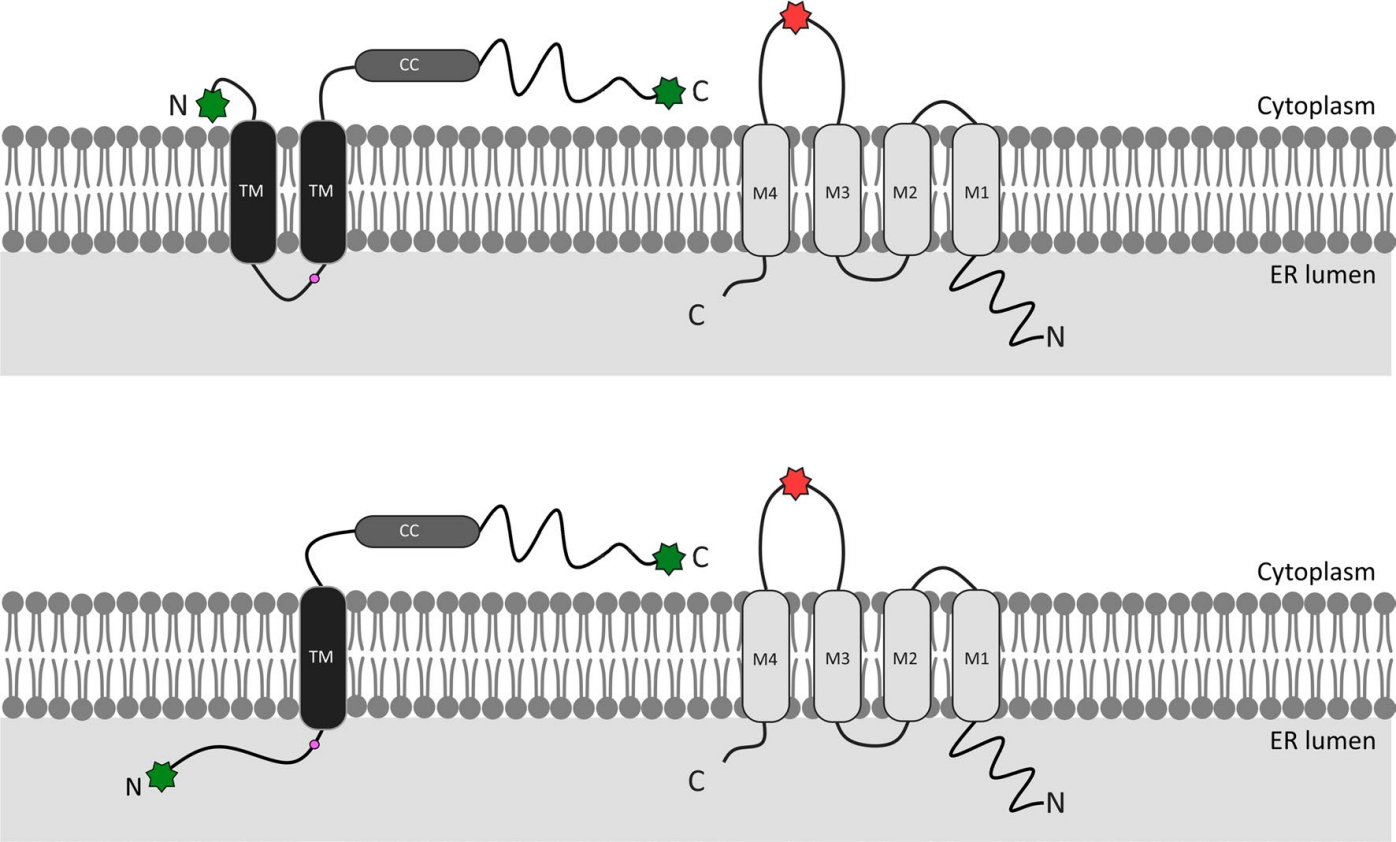
Schematic showing proposed topology and tagging of α7 and RIC3. **A**: Two transmembrane (TM) domains result in cytoplasmic C- and N-termini. **B**: A single transmembrane domain results in a cytoplasmic C-terminal domain and a lumenal N-terminal domain. Red star denotes tagging of α7 subunit between the TM3 and TM4 domains. Green stars represent tagging of RIC3, Pink dot denotes position of G88R variant. CC denotes coiled-coil domain

Given that cell surface expression is a pre-requisite for α7 nAChR signaling, the identification of the structural domains involved in the chaperone activities of RIC3 has been a long-standing research goal. RIC3 is a disordered protein with little homology between species [17]. Its structural domains comprise an N-terminal region that contains two hydrophobic segments linked by a proline-rich linker and a long C-terminal region that contains either one (human, mouse, *Drosophila*) or two (*C. elegans*) coiled-coil motif [19] (Fig. 1). The G88R variant identified in the backward speech study occurs within a poly-glycine stretch found inside the proline-rich linker domain (Fig. 1). For invertebrate species, the two hydrophobic segments are predicted to be transmembrane domains. In contrast, for mammalian species, the location of the segments is controversial. Wang et al. working with mouse RIC3 identified a cleavable signal peptide in the N-terminus, which led to the suggestion that RIC3 is a single-pass type I transmembrane protein with its N-terminus located in the lumen of the ER and the C-terminus with its coiled-coil domain in the cytoplasm [17]. In the human RIC3, Cheng et al. [39] reported a cleavable sequence in the N-terminus of human RIC3 [39], but others found no evidence the N-terminus is cleaved during translation [25, 40]. Our findings suggest that human RIC3 is a type II transmembrane protein with the N- and C-termini facing the cytoplasm (Fig. 1). The latter topology is consistent with the findings that the complete N-terminus is crucial for efficient cell surface expression of invertebrate and mammalian α7 nAChR [17, 18, 22, 25, 40].

The exact mechanism by which RIC3 promotes α7 nAChR assembly is unknown [41] although a direct interaction is expected as α7 co-precipitates with RIC3 [20, 42]. Wang et al. suggested that each RIC3 protein associates with a single folded α7 subunit [17]. The receptor is then built through RIC3 dimerization at the C-terminal coiled-coil motif, pulling subunits together to form the pentamer [17]. However, others note that the coiled-coil domain is not required for RIC3 function [40, 43] and that some isoforms of RIC3 lack the coiled-coil domain but are still able to promote α7 assembly [18, 22]. Ben-David et al. further showed that the shorter isoform, which lacks the coiled-coil domain, has different functional properties from the full protein and acts as an inhibitor of AChR assembly and function [22]. Kweon et al. later suggested that the α7 assembly process involves a host of chaperone proteins, including NACHO, OST, RPN1/2, and calnexin, as well as RIC3 passing α7 through the secretory pathway [44]. Each of these chaperones is thought to bind a distinct region of α7 [44] and it has been proposed that RIC3 binds between the M3 and M4 transmembrane domains [44]. This loop is largely disorganized but includes an MX helix and an MA helix, that runs into the M4 transmembrane domain [41]. Although studies have shown that this region is necessary for the effects of RIC3 [44] and that substitution of residues in the MA helix ablates RIC3 enhancement of assembly [25], structural modeling suggests that interactions in this region would block pentameric assembly [41].

In the present study, we examined the consequences of G88R variant on interactions between RIC3 and α7 nAChR subunits in HEK293 mammalian cells, using RIC3 and α7 nAChR tagged with fluorescent proteins. Led by previous research, we examined three levels of function; the cellular localization of the RIC3, interactions between RIC3 and α7, and the surface expression of mature α7 receptors. Using acceptor photobleaching (ap) FRET, we found that G88R increases interaction between RIC3 and α7 in the ER. Interestingly, we found that the enhanced interaction results in decreased functional expression of α7 nAChR in *Xenopus* oocytes and reduced ^125^I-α-bungarotoxin binding in HEK293 cells. We suggest that the G to R variant exerts a functional effect through increased interaction between α7 nAChR and RIC3 in the ER, ultimately leading to reduced functional expression.

This investigation not only establishes a functional effect for the G88R variant but provides additional evidence on the structure of RIC3 and the mechanism of interaction between RIC3 and α7.

## Methods

### Reagents

^125^I-α-Bungarotoxin (NEX126H050UC) was obtained from PerkinElmer, UK. Fugene was obtained from Promega (E5911). Acetylcholine (A2661), polyethylenimine, 25,000 MW (4008727), and Triton (648466) were obtained from Merck.

### Cell culture and cell transfections

Human Embryonic Kidney 293 cells (HEK293, supplied by ATCC, UK) were cultured in DMEM (1X) with high glucose (Life Technologies, UK) supplemented with 10% fetal calf serum (FCS; Life Technologies, UK). Cells were used for experimentation once they reached 60–70% confluency. Cells were plated on poly-d-lysine (0.1 mg/ml, Sigma)-coated glass-bottomed μ-dish 35 mm Ibidi dishes (Thistle Scientific), UK at a density of 120,000 cells/ml. All cultures were maintained at 37 °C and 5% CO_2_.

For confocal microscopy, HEK293 cells were transfected (0.5 µg of α7, RIC3, LCK, and ER3 plasmids) using FuGene HD (Promega) following manufacturer’s instructions.

### Constructs

#### α7 clone

Wild-type human α7 nAChR subunits were synthesized by GeneArt (ThermoFisher, UK). The sequence of the cDNA was optimized for expression in mammalian cells. Fluorescently tagged α7 nAChR subunits were produced by inserting mCherry cDNA into the M3-M4 cytoplasmic loop of α7 at amino acid 391 (α7-mCherry).The positioning of the tag has previously been demonstrated to retain the functional properties of the receptor [45] and sits 74 amino acids away from the MX helix, which is proposed to be the site of interaction between RIC3 and α7 [46, 47]. Both wild-type and fluorescent α7 nAChR subunit cDNAs were subcloned into the pCI expression vector (Promega, UK).

#### RIC3 clones

Wild-type RIC3, henceforward termed RIC3WT, cDNA (NM_024557) was amplified using primers containing EcoRI (5’ TATTCGAATTCGCGTACTCCACAGTGCAGAGAGTCGCTCTGG 3’) and KpnI (5’ AATAAGGTACCTCACTCTAAACCCTGGGGGTTACGCTTCCTCAG 3’) restriction sites. Site-directed mutagenesis (F-primer 5’ AGGTGGAGGTGCTGGACGTGGAGGTAGTGGAAGAGG 3’, R-primer 5’ CCTCTTCCACTACCTCCACGTCCAGCACCTCCACCT 3’) was used to introduce the G88R variant (NM_024557.4:c.262G > A, NP_078833.3:p.G88R) into RIC3 (RIC3G88R).

RIC3WT and RIC3G88R cDNAs were subsequently cloned into the MCS of pEGFP-N1 (NovoPro Bioscience, Shanghai, China) to fuse the fluorescent eGFP tag to the C-terminus of RIC3WT (RIC3WT-eGFP) or RIC3G88R (RIC3G88R-eGFP) or pEGFP-C1 (NovoPro Bioscience, Shanghai, China) to fuse the eGFP tag to the N-terminus of the RIC3 protein (eGFP-RIC3WT and eGFP-RIC3G88R).

### Western blots

HEK293T cells were seeded at 3.5 × 10^6^ cells per 10 cm plate and transfected with 17.5 μg plasmid (eGFP-RIC3WT or eGFP-RIC3G88R). After 48 h, protein lysates were extracted and quantified using a BCA assay. Proteins were separated on a 10% SDS-PAGE gel for 30 min before transfer to a nitrocellulose membrane using a semi-dry protocol for high MW proteins. Membranes were blocked in 5% milk powder in 1 × TBS Tween before detection with primary rabbit polyclonal antibodies for GFP (AbCam; ab290) and secondary goat anti-rabbit IgG (Licor.com; IRDye 680RD) for RIC3 detection. A primary mouse monoclonal antibody for α-tubulin (Merck; T5168) with secondary anti-mouse IgG (Licor.com; IRDye 800CW) was used as a positive control. All antibodies were used at a 1 in 1000 dilution. Membranes were washed six times with 1 × TBS Tween and visualized on a Typhoon biomolecular imager (Cytiva) against a marker precision plus ladder (Biorad; 161-0374).

### Confocal microscopy and acceptor photobleaching FRET

Acceptor photobleaching fluorescence resonance energy transfer (apFRET) [48, 49] was used to detect interactions between the tagged α7 and RIC3 proteins using a Zeiss LSM880 confocal microscope 2 days after transfection.

eGFP was used as the FRET donor and mCherry as the FRET acceptor. pmCherry-eGFP (Addgene, plasmid#86639) was used as positive control, while mCherry-ER3 (Addgene, plasmid#55041) and LCK-GFP (Addgene, plasmid#61099) were used as negative controls for RIC3-eGFP and α7-mCherry, respectively. FRET between donor and acceptor was confirmed by bleaching of mCherry which lasted 5 s and monitoring the concomitant increase in eGFP fluorescence across five successive 0.47 s windows. mCherry was excited with 561 nm light and eGFP with 488 nm light. The mCherry and eGFP laser transmission was kept at 2% and 1.5%, respectively, during scanning to avoid photobleaching but mCherry was set at 100% during bleaching. HEK293 cells expressing either eGFP or mCherry alone were imaged with the apFRET settings to confirm that fluorophore crosstalk was minimized, and that the bleaching step did not reduce eGFP fluorescence. Five pre-bleach and five post-bleach scans of the eGFP and mCherry fluorescence were carried out at 0.47 s intervals in a constant sized region of interest (ROI) which was manually selected to represent an ER location with comparable levels of red and green fluorescence. Fluorescence intensity was monitored in the ROI and analyzed using Microsoft Excel. For data analysis, the eGFP fluorescence intensity was normalized onto a percentage scale as described previously [48, 49]. To calculate the FRET efficiency *E*_F_, the following equation was used, as described by Graumann et al. [49]:

$$E_F = {\text{eGFPpost}} - {\text{eGFPpre}}$$

where eGFPpost is the fluorescence intensity immediately after the photobleaching (scan 6) and eGFPpre is the average fluorescence intensity across all five scans before the photobleaching. Note that this calculation assumes 100% photobleaching of the acceptor [50]. All confocal work was performed at the Oxford Brookes Centre for Bioimaging. For each experimental and control sample, approximately 100 live cells (in DMEM) were imaged with a 63 × oil immersion objective (Plan-Apochromat 63×/1.4 Oil DIC M27) at 37 °C and 5% CO_2_. Each experiment was repeated three times. After excluding outliers (> ± 1.5(IQR)), the number of intensity measurements included in the FRET calculation for each condition were *N* = 246 (α7-mCherry + eGFP-RIC3WT), *N* = 257 (α7-mCherry + eGFP-RIC3G88R), *N* = 233 (α7-mCherry + LCK-GFP, negative control), *N* = 239 (eGFP-RIC3WT + mCherry-ER3, negative control), *N* = 231 (eGFP-RIC3G88R + mCherry-ER3, negative control), *N* = 244 (pmCherry-ER3-eGFP, positive control).

### ^125^I-α-Bungarotoxin binding

RIC3WT or mutant RIC3G88R cDNA, in combination with α7 cDNA (at a ratio of 1:5 or 1:1), were transfected into HEK293 cells using polyethylenimine. Surface α7 expression was determined 48 h after transfection by overlaying the cells in phosphate-buffered saline (PBS) containing 10 nM ^125^I-α-bungarotoxin and 1 mg/mL bovine serum albumin for 60 min. Cells were washed four times with PBS and removed from the plate in 10 mM Tris–HCl (pH 7.4), 100 mM NaCl, 1 mM ethylenediaminetetraacetate, and 1% Triton X-100. ^125^I-α-Bungarotoxin binding was determined by gamma counter.

### Functional expression of nAChR in Xenopus oocytes

Electrophysiological experiments were carried out on oocytes nuclearly injected with either α7 cDNA, RIC3WT, RIC3G88R, α7 + RIC3WT or α7 + RIC3G88R. We also tested the effects of wild-type and variant RIC3 on the functional expression of human α4β2 nAChRs. For these experiments, oocytes were nuclearly injected with equal amounts of α4 and β2 cDNA. For both types of injections (α7and α4β2 nAChR subunit cDNAs), the total amount of cDNA injected was kept at 5 ng for nAChR subunit cDNA and 1 nG for RIC cDNAs. Oocytes were harvested from mature *Xenopus laevis* females and used for electrophysiological experiments 2 days after injection, as described previously [51].

Acetylcholine-induced currents in Xenopus oocytes expressing heterologously α7 nAChR were recorded using an automated platform equipped with standard two electrode voltage-clamp configuration (HiClamp; Multi Channel Systems, Reutlignen, Germany). The electrodes were filled with 3 M KCl and the recordings were carried at a holding potential of − 60 mV throughout the experiment. All recordings were performed at 18 °C, and cells were perfused with a solution containing 82 mM NaCl, 2 mM KCl, 2 mM CaCl_2_, 5 mM HEPES at pH 7.4. Data were filtered at 10 Hz, captured at 100 Hz, and analyzed using proprietary data acquisition and analysis software running under Matlab (Mathworks Inc., Natick, MA). Maximal functional expression was determined using 1 mM acetylcholine, a concentration that produces maximal current responses at oocytes expressing α7 nAChRs [51]. The concentration–response curve for acetylcholine at α7 nAChR was also determined to establish whether the variant affected the function of α7 nAChR. For these experiments, we used a protocol of 7–8 concentrations of acetylcholine with a reference response (1 mM ACh, a maximal ACh concentration in wild-type human α7 nAChR). Acetylcholine was applied for 10 s and the washing period between applications was 5 min to allow for full recovery from receptor desensitization [51]. The concentration–response data were fit with the Hill equation to estimate the acetylcholine potency (ACh EC_50_), as previously described [51]. For α4β2 nAChR assays, functional expression of α4β2 nAChR in oocytes injected with α4 and β2 cDNAs ± WT RIC3 or RIC3G88R cDNA was assessed by measuring the amplitude of current responses elicited by application of a maximal ACh (1 mM) to the impaled oocytes. For these assays, currents were recorded using an oocyte clamp OC-725Camplifier (Warner Instruments). For all receptor subtypes assayed and examined, the current responses to ACh were recorded 2 days after injection and all experimental conditions (RIC3WT or RIC3G88R) were done on the same day.

### Image processing and statistical analysis

**Fig. 2:**
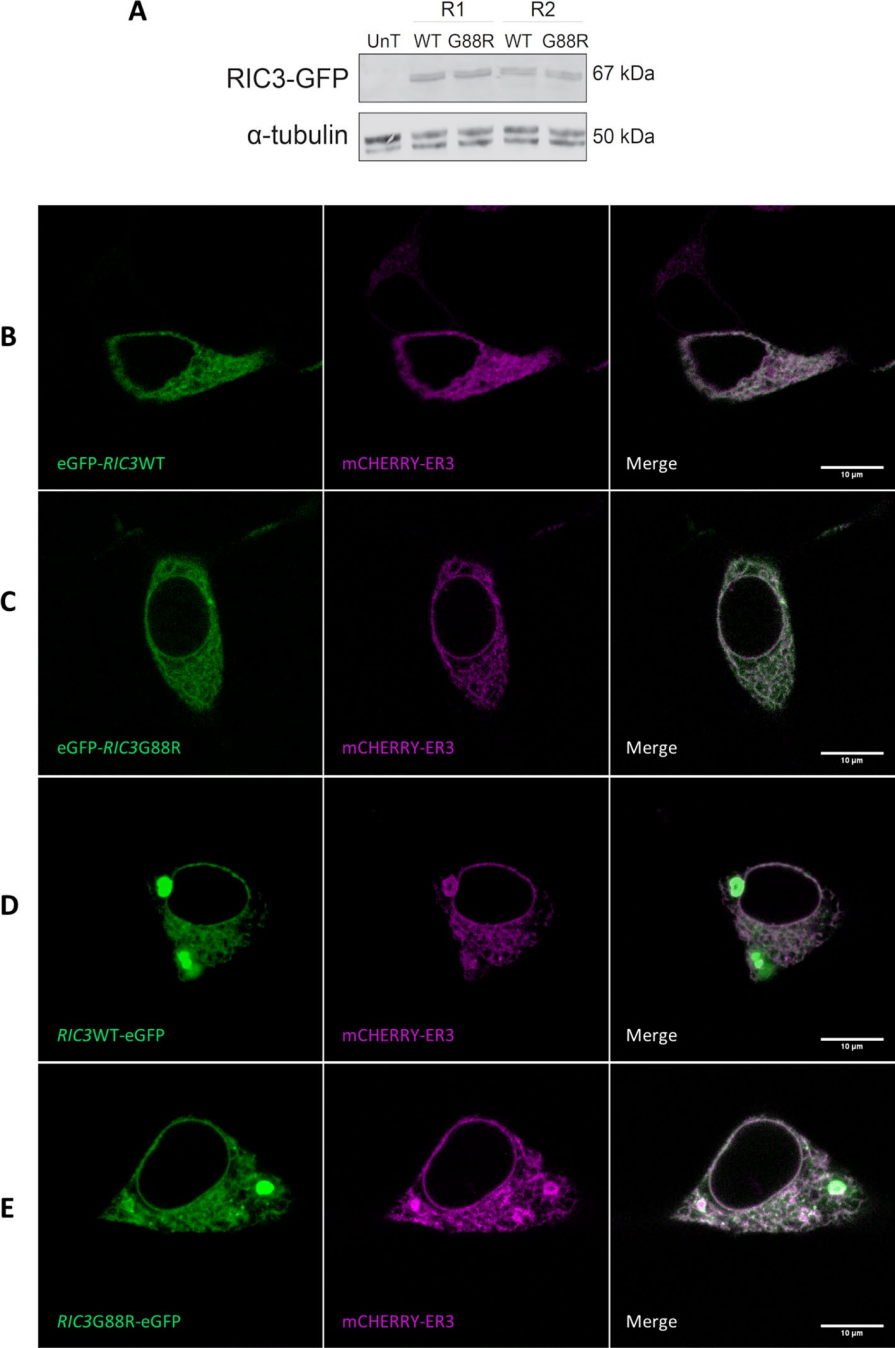
Cellular localization of RIC3WT and RIC3G88R. **A**: Western blot of transfected cells showed the presence of an eGFP-RIC3 protein at the expected 67KDa size (UnT—untransfected HEK293 cells).Two biological replicates were performed (R1 and R2). No observable differences were present between wild-type (WT) and variant (G88R) cell-lines. **B** and **C**: N-terminal fusion of eGFP on wild-type (eGFP-RIC3WT) and G88R (eGFP-RIC3G88R). **D** and **E**: C-terminal fusion of eGFP on wild-type (RIC3WT-eGFP) and G88R (RIC3G88R-eGFP) Formation of RIC3 bright oval structures was observed in the ER. RIC3 demonstrated a strong overlap with the ER marker (mCherry-ER3) in both the wild-type (WT) and variant (G88R) forms and for both N-terminal and C-terminal fusions. The average Pearson correlation coefficient (PCC) across five representative images for eGFP-RIC3WT + mCherry-ER3 (Panel **B**) was 0.73 (SD = 0.12), for eGFP-RIC3G88R + mCherry-ER3 (Panel **C**) was 0.67 (SD = 0.08), for RIC3WT-eGFP + mCherry-ER3 (Panel **D**) was 0.61 (SD = 0.14) and for RIC3G88R-eGFP + mCherry-ER3 (Panel **E**) was 0.78 (SD = 0.11). Size bars = 10 µm. Raw Western blot images are provided in supplementary data

**Fig. 3:**
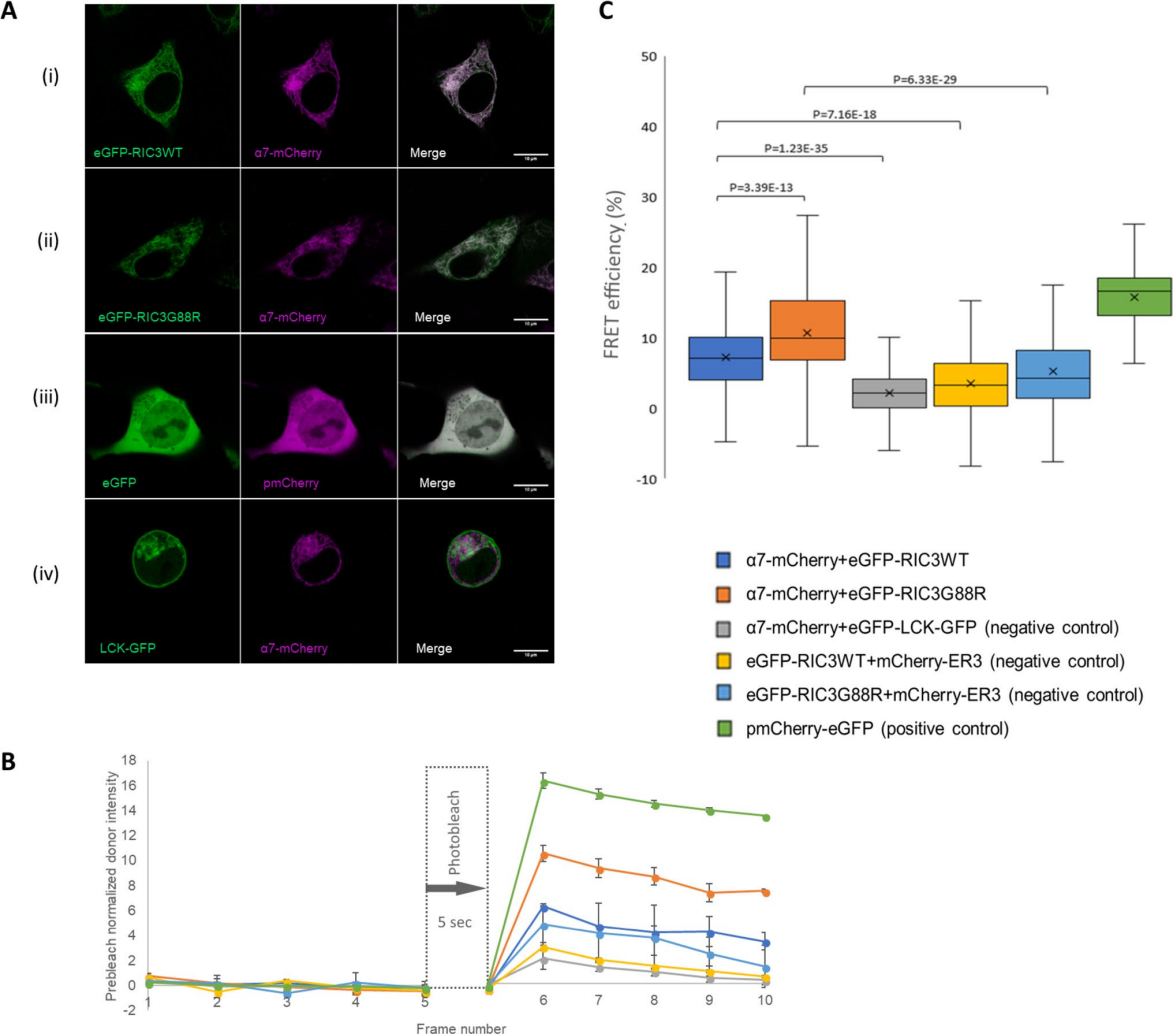
Interaction between α7 and eGFP-RIC3WT or eGFP-RIC3G88R measured by apFRET. **A**: Representative confocal images of the proteins of interest (α7 and RIC3WT (Panel i), α7 and RIC3G88R (Panel ii)). eGFP was used as the FRET donor and mCherry as the FRET acceptor. pmCherry-eGFP (Addgene, plasmid#86639) was used as positive control (Panel iii), while LCK-GFP (Addgene, plasmid#61099) and α7-mCherry were used as negative controls (Panel iv). **B**: Normalized donor and acceptor fluorescence intensity post-acceptor photobleaching. Each interval is 0.47 s. Photobleaching occurred across 5 s at time interval 5. Error bars represent SD across all measurements within each of the experimental conditions. See methods for details of normalization and N. **C**: FRET efficiencies measured for α7 and eGFP-RIC3WT or eGFP-RIC3G88R and controls, as described in methods. Boxes represent interquartile range of FRET intensity (see Methods for *N*), with lines at the median and crosses denoting mean of distribution. RIC3 demonstrated a strong overlap with α7 in both the wild-type (WT) and variant (G88R) forms. The average Pearson correlation coefficient (PCC) across five representative images for eGFP-RIC3WT + α7-mCherry (Panel A(i)) was 0.70 (SD = 0.08) and for eGFP-RIC3G88R + α7-mCherry (Panel A(ii)), the average PCC was 0.72 (SD = 0.04). The positive controls (pmCherry-eGFP) also showed a strong co-localization (Panel A(iii))—PCC across five representative images was 0.82 (SD = 0.09), while the negative controls (LCK-GFP + mCherry) had minimal overlap (Panel A(iv))—PCC across five representative images was 0.26 (SD = 0.11). Size bars = 10 µm

Images were analyzed within Fiji [52] to assess co-localization of proteins. Images were imported as raw.czi files and a single timepoint was extracted for the red and green channels. All images were subject to background subtraction using sliding paraboloid method with a rolling ball of radius 50 pixels. Co-localization analyses were performed on a region of interest that included the whole cell using a Coloc2 plugin (https://imagej.net/plugins/coloc-2). 2D intensity histograms for the representative images shown in Figs. 2 and 3 are provided as Supplementary data. Co-localization is reported as Pearson correlation coefficients (PCC) throughout.

Unadjusted representative images were exported as montages of raw files in which red was replaced with magenta. Brightness and contrast were adjusted for all channels simultaneously in PowerPoint.

Data are expressed as means ± SEM from 100 experiments carried out using 12–14 batches of transfected cell batches or ten *Xenopus* donors. Data are reported as mean ± SEM. To compare significant differences (at* p* $<$ *0.05*) between more than two groups of data meeting assumptions of normality and equal variance, a one-way ANOVA was performed followed by a Tukey test for all pair-wise comparisons.

## Results

### RIC3G88R localization

Previous studies consistently report that RIC3 localizes to the ER, where it binds unassembled α7 subunits promoting receptor assembly [17, 21, 53]. Therefore, we first sought to assess whether the G88R variant affected RIC3 cellular localization. Both RIC3WT and RIC3G88R were fused to eGFP at the N-(eGFP-RIC3WT and eGFP-RIC3G88R) or C-(RIC3WT-eGFP and RIC3G88R-eGFP) terminus and transiently expressed in HEK293 cells.

All four RIC3 clones co-localized with mCherry-ER3, an ER-resident protein marker (Fig. 2b–e, see Figure legends for Pearson Correlation Coefficients) confirming an ER localization for RIC3 and indicating that the G88R variant does not overtly alter cellular localization (Fig. 2b–e). In addition to the ER localization, both RIC3WT-eGFP and RIC3G88R-eGFP also resulted in bright ring structures (Fig. 2d and e). Similar bodies were observed by Wang et al. using mouse *Ric3*, who suggested that this pattern may arise from over-expression leading to homotypic interactions between *Ric3* [17]. In addition, it was observed that both RIC3WT-eGFP and RIC3G88R-eGFP transfections led to a distorted ER structure (Fig. 2d and e), further indicating disruption of the secretory pathway. eGFP-RIC3WT (Fig. 2b) and eGFP-RIC3G88R (Fig. 2c) were, therefore, used for the remainder of experiments in this paper. Western blots confirmed that the full-length eGFP-RIC3 protein was present at 67KDa as expected (Fig. 2a).

### Interaction between α7 and RIC3

To obtain direct evidence whether the RIC3 variant affected interaction with α7, acceptor photobleaching fluorescence resonance energy transfer (apFRET) was employed (Fig. 3). This method is based on the fact that when energy transfer occurs, the fluorescence emission by the donor fluorochrome is quenched because of the direct transfer of excitation energy to the acceptor fluorochrome. If the acceptor fluorochrome is fully bleached by a laser, FRET is dampened and the donor signal is de-quenched, thus resulting in an enhanced fluorescence emission by the donor fluorophore [21, 54]

α7 was labeled with mCherry and was observed to co-localize with eGFP-RIC3 (Fig. 3, see Figure legend for Pearson correlation coefficients). FRET efficiency between α7 and eGFP-RIC3G88R, *E*_F_ = 10.73% ± 7.06, *N* = 100, was significantly higher (*p* < 0.05; ANOVA plus Tukey test) than that observed for eGFP-RIC3WT (*E*_F_ = 7.24% ± 5.97, *N* = 100) (Fig. 3). Furthermore, the fluorescent signal emitted by the donor (eGFP) fluorochrome as a result of dequenching was 1.5-fold higher in cells carrying the variant compared to wild type (*N* = 30; *p* < 0.05; ANOVA plus Tukey test) (Fig. 3b and c) and this change persisted over the duration of the experiment (Fig. 3b). These results collectively suggest that the G88R SNP enhances interactions between RIC3 and α7 nAChR subunits.

### Cell surface expression

**Fig. 4:**
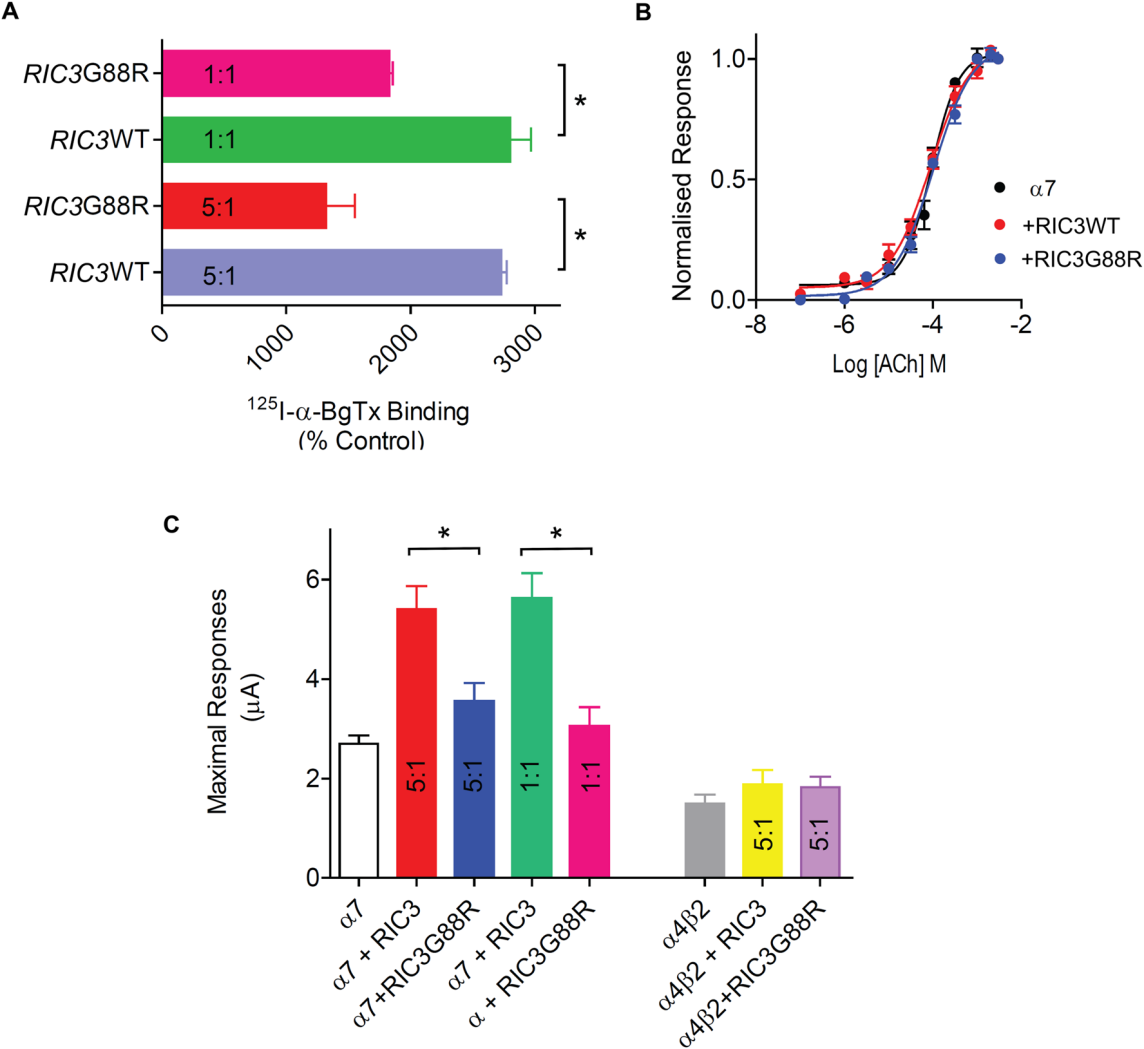
RIC3G88R variant decreases cell surface expression of α7 nAChR. **A** Cell surface expression of α7 nAChRs was assayed by ^125^I-α-bungarotoxin binding to intact HEK293 cells transfected with mCherry α7 and either RIC3WT or RIC3G88R at 5:1 or 1:1 cDNA ratio. **B**: The potency with which acetylcholine activates responses in α7 nAChRs is not affected by RIC3WT or the variant RIC3G88R (*N* = 5). **C**: Histograms of the maximal currents activated by 1 mM Ach in α7 (*N* = 15) and α4β2 (*N* = 10)

To evaluate the effect of the enhanced interaction between α7 and RICG88R on cell surface expression, we performed ^125^I-α-bungarotoxin binding on intact HEK293 cells co-transfected with mCherry α7 and eGFP-RIC3WT or eGFP-RIC3G88R. The cDNAs were transfected at either 1:1 or 5:1 α7:RIC3 ratios. α-Bungarotoxin is a neurotoxin that binds competitively to the agonist binding site of α7 nAChR subunits. The agonist binding site in nAChR is located between two adjacent subunits; hence, ^125^I-α-bungarotoxin binding to cells transfected with α7 cDNA can be used to probe receptor assembly. Cells transfected with α7 nAChR cDNA did not bind ^125^I-α-bungarotoxin (data not shown). In contrast, in the presence of RIC3, robust specific ^125^I-α-bungarotoxin binding was observed (Fig. 4). As shown in Fig. 4a, regardless of the transfection ratio, RIC3G88R significantly decreased the binding of ^125^I-α-bungarotoxin (*N* = 6; *p* < 0.05; ANOVA) by almost 50%, suggesting that enhanced RIC3-α7 interactions decrease cell surface expression of α7 nAChR. The reduction of ^125^I-α-bungarotoxin binding observed with transfection ratios of 1:1 or 5:1 was not statistically different to each other, in accordance with the findings of Dau et al., who reported that RIC3 significantly enhanced cell surface α-bungarotoxin binding above control levels at both 1:1 and 5:1 α7:RIC3 ratios [21].

Next, we examined whether the reduced cell surface expression of α7 nAChR affected α7 function. To examine function, α7 was expressed in the absence or presence of untagged RIC3WT or RIC3G88R in *Xenopus* oocytes, a well-established expression system ideally suited for electrophysiological recordings of recombinant ion channels. The nucleus of oocytes was injected with α7:RIC3 cDNA at a ratio of 1:1 or 5:1, and the amplitude of currents generated by 1 mM acetylcholine was recorded. At this concentration, acetylcholine stimulates maximal current responses in α7 nAChR, which are indicative of the level of functional receptors present. As shown in Fig. 4b, functional expression of α7 nAChR in the absence or presence of RIC3 does not impact the potency of Ach, as previously reported [55]. Regardless of the α7:RIC3 cDNA ratio, in the presence of RIC3WT, the expression of functional α7 nAChR increased by approximately twofold (Fig. 4c, *N* = 15, *p* < 0.001), further supporting the ability of this chaperone to promote surface expression in *Xenopus* oocytes. Consistent with the findings of the binding studies, the levels of functional α7 nAChR decreased by about 1.5-fold in the presence of RIC3G88R (Fig. 4c, p < 0.05). Tagged α7 and RIC3WT or RIC3G88R produced the same pattern as the non-tagged constructs (data not shown). In addition, we also examined the effect of RIC3WT or RIC3G88R on the functional expression of human α4β2 nAChR. As shown in Fig. 4c, under our experimental conditions (5 ng of α4 + β2 cDNA mixture ± 1 ng RIC cDNA), both RIC3WT and RIC3G88R increased the expression of α4β2 nAChR similarly; however, none of these effects were statistically significant compared to control (α4β2) (*N* = 10 recordings from two oocyte donors). Previous studies have shown that the effect of RIC3 on functional expression of α4β2 nAChR is not consistent, suggesting that, in oocytes, the effects may depend on other elements expressed in oocytes [18, 19, 21]. Thus, RIC3G88R appears to selectively impact the functional expression of α7 nAChR.

## Discussion

While several general protein chaperones modulate the maturation and trafficking of nAChRs [56], RIC3 is relatively specific in its chaperone activity exerting significant effect upon the folding and assembly of α7 receptors [20]. Interestingly, a rare genetic variant of RIC3 (NP_078833.3:p.G88R) was potentially implicated in a unique ability to speak backwards that is associated with higher working memory capacity [38]. This variant was one of three novel coding changes that co-segregated with the trait in the discovery family but the authors particularly highlighted the *RIC3* polymorphism, hypothesizing that this may exert a function upon cholinergic systems [38]. In this investigation, we, therefore, sought to establish the functional level of effects mediated by this coding change in *RIC3*. We find that RIC3G88R significantly increased interaction with α7 compared to the wild-type RIC3. Subsequent ^125^I-α-bungarotoxin binding to α7 and functional assays showed that RIC3G88R decreased cell surface binding and functional expression, suggesting that enhanced RIC3-α7 interactions in the ER reduce cell surface and functional expression of α7 nAChR. This finding indicates that the polymorphism RIC3G88R modifies RIC3-α7 interactions and that this change substantially affects α7 nAChR surface expression. The exact relationship between this functional pathway and backwards speech is still to be elucidated. Many questions remain regarding the way in which RIC3 moderates receptor assembly and function and whether these effects are specific to certain receptor types. Understanding these mechanisms will be critical to the functional characterization of this variant, which may act at many different levels.

Our investigations show that eGFP-RIC3WT produced a significantly higher FRET signal/efficiency compared to the negative controls. A previous study also used FRET to demonstrate increased assembly and cell-surfacing trafficking of α7 in the presence of RIC3 [21]. However, this investigation used a different FRET method (sensitized emission) and measured interaction between α7 subunits [21]. Others have shown co-immunoprecipitation of RIC3 and α7 [16]. Our findings add to this baseline to further suggest a direct interaction between RIC3 and α7 within the ER. Interestingly, the FRET signal produced by eGFP-RIC3G88R was almost 1.5-fold greater than that seen with eGFP-RIC3WT. This strongly indicates that there is a direct interaction between RIC3 and α7 and that the p.G88R polymorphism implicated in backwards speech strengthens this interaction, resulting in a reduced surface expression, as shown by ^125^I-α-bungarotoxin binding and the decrease in the amplitude of the maximal currents elicited by Ach in oocytes expressing heterologously α7 nAChR and RIC3G88R. Although the role of RIC3 in α7-signaling dysfunction has not been explored, α7 nAChR expression is reduced in the brain of schizophrenic patients [5, 57] and the levels of RIC3 mRNA in the brains of schizophrenia patients, postmortem, are greater than in typical brains [26]. Chaperones of nAChR have been previously linked to cholinergic dysfunction. A variant of rapsyn, a muscle nAChR chaperone that concentrates and anchors muscle nAChR in the postsynaptic membrane of the neuromuscular junction, causes congenital myasthenic syndrome by altering interactions with the receptor muscle specific tyrosine kinase (MuSK) [58].

Variant G88R occurs within a poly-glycine stretch found within the proline-rich linker that joins the hydrophobic domains of RIC3. Deletion of the entire proline-rich linker in human RIC3 [25] attenuates α7 surface expression, indicating the importance of this region for the chaperone activity of RIC3, although specific singular residues are unlikely to account for this effect. The poly-glycine segment is not thought to adopt a specific folding pattern but we propose that the G-R change creates a positively charge that may alter the configuration of the proline region, thus affecting chaperone activity of RIC3.

We found that N-terminal fusion of wild-type RIC3 to eGFP (eGFP-RIC3) does not impair the expression of RIC3 in the ER or the chaperone activity of this protein. These findings are in accord with previous studies of human RIC3 that have used N-terminal fusion RIC3 constructs to examine the chaperone activity of this protein [21, 53]. The observation of FRET activity between eGFP-RIC3 and α7 directly indicates that RIC3 is a type II transmembrane protein, since the N-terminus of RIC3 must have a cytoplasmic location to allow this interaction (Fig. 1a). This supposition opposes the findings of Wang et al. who working with a C-terminal tagged mouse RIC3 construct suggested that the mouse Ric3 N-terminus is cleaved during translation [17].

In contrast, the expression of the C-terminal fusion, RIC3-eGFP, led to rings of bright fluorescence and a disordered ER, reminiscent of ER-phagy [59]. These observations suggest that tagging of the C-terminus disrupts RIC3 function leading to misfolded polypeptides within the ER and subsequent removal of damaged ER sections. If the misfolded proteins are α7, then this implicates a direct role for RIC3 in α7 folding as suggested by [17]. Given the disordered nature of RIC3, the localization of the exact interaction domain has proven to be challenging [41].

In summary, our investigations shed light upon the interaction between RIC3 and α7 in the assembly and trafficking of this important neuronal receptor. Specifically, we demonstrate that the RIC3G88R variant has a functional effect by increasing RIC3 interaction with α7 subunits in the ER and that this ultimately leads to a reduction in the cell surface and functional expression of α7 nAChR. How may a decrease in α7 receptor expression influence the ability to speak backwards? Backward speech relies on a strong working memory capacity [38] and memory is influenced by α7 nAChR signaling [60]. The role of α7 nAChR in cognition is linked to its modulation of glutamatergic and GABAergic signaling but the mechanisms driving this effect are not well-understood. This is largely due to the complexity of α7-signaling, which is affected by diverse elements including cell type, location, and complex relationship between timing of activation relative to associated glutamatergic and GABAergic pathways involved in cognition and memory (for a review, see [61]). Thus, decreased functional expression of α7 nAChR could potentially upset the balance between the modulation of excitatory/inhibitory pathways. Alternatively, decreased expression of α7 nAChR may alter neuronal development, when the foundations for the cognitive and language functions of the brain are first laid. ^125^I-α-Bungarotoxin binding sites are present in the human fetal brain [62], and the α7 nAChR has been implicated in neuronal migration [63] and early post-natal synapse formation [64, 65]. Thus, RIC3 can potentially affect the ability to speak backwardly by affecting the establishment of the signaling circuitries involved in speech. Further investigations will be required to link this functional finding to the reported language phenotype, providing important evidence about the function of both RIC3 and α7 nAChR in neurodevelopment.

### Supplementary Information

Below is the link to the electronic supplementary material.

Supplementary file1 (PDF 1486 KB)

## Data Availability

The datasets generated during and/or analyzed during the current study are available in the Oxford Brookes RADAR repository [10.24384/mp84-n694].

## Abbreviations

apFRET: Acceptor photobleaching fluorescence resonance energy transfer
eGFP: Enhanced green fluorescent protein
ER: Endoplasmic reticulum
FRET: Fӧrster resonance energy transfer
nAChR: Nicotinic acetylcholine receptor
MCS: Multiple cloning site
mCherry: Monomeric cherry fluorescent protein
