# Supplementary material for: Unraveling the molecular interactions between α7 nicotinic receptor and a RIC3 variant associated with backward speech
Source: Cell Mol Life Sci. 2024 Mar 12;81(1):129. doi: 10.1007/s00018-024-05149-8 (PMC10933150; doi:10.1007/s00018-024-05149-8)

Supplementary Data - 2D Histogram plots for images shown in Figures 2 and 3

2D intensity histogram
256.0 x 256.0

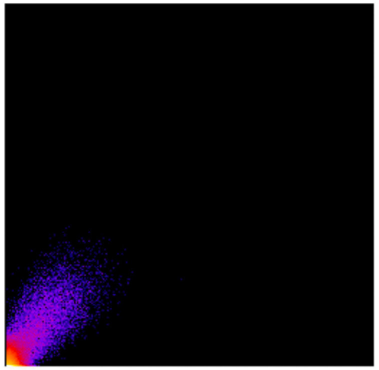

eGFP-RIC3WT+mCherry-ER3
Fig 2. Panel A

2D intensity histogram
256.0 x 256.0

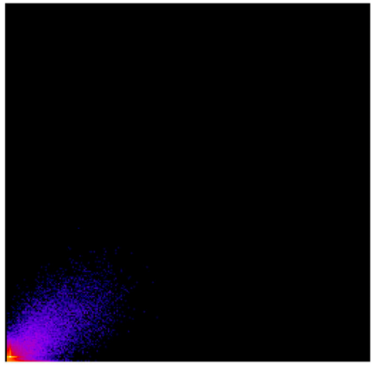

eGFP-RIC3G88R+mCherry-ER3
Fig 2. Panel B

2D intensity histogram
256.0 x 256.0

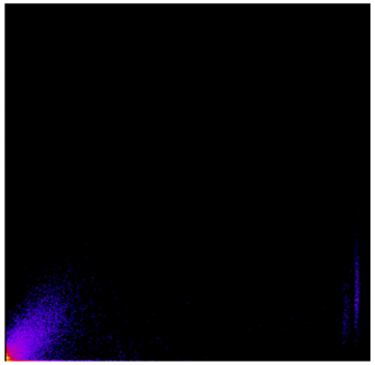

RIC3WT-eGFP+mCherry-ER3
Fig 2. Panel C

2D intensity histogram
256.0 x 256.0

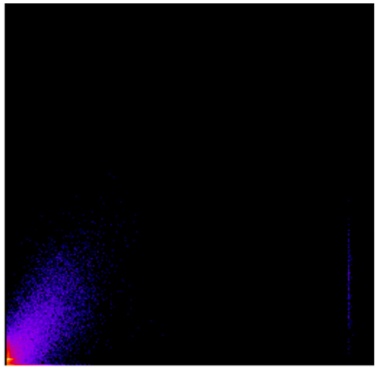

RIC3G88R-eGFP+mCherry-ER3
Fig 2. Panel D

2D intensity histogram
256.0 x 256.0

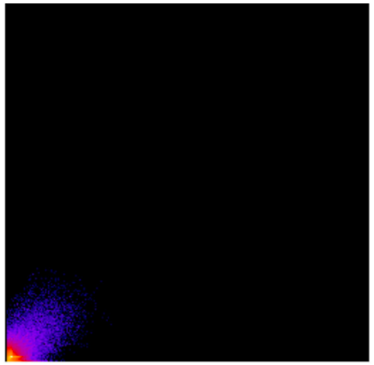

eGFP-RIC3WT+α7-mCherry
Fig 3. Panel A(i)

2D intensity histogram
256.0 x 256.0

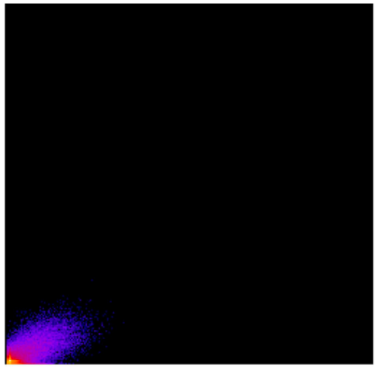

eGFP-RIC3G88R+α7-mCherry
Fig 3. Panel A(ii)

2D intensity histogram
256.0 x 256.0

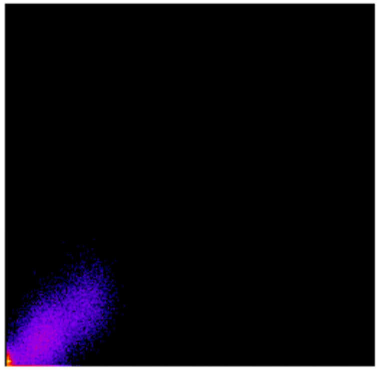

pmCherry-eGFP
Positive control
Fig 3. Panel A(iii)

2D intensity histogram
256.0 x 256.0

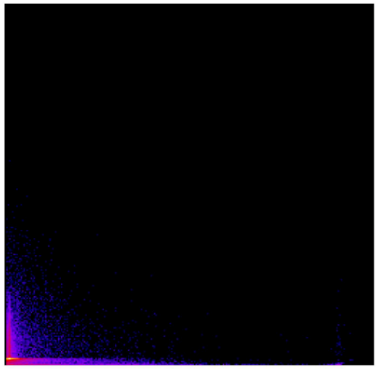

LCK-GFP+α7-mCherry
Negative control
Fig 3. Panel A(iv)

Supplementary Data – Raw Western Blots

A

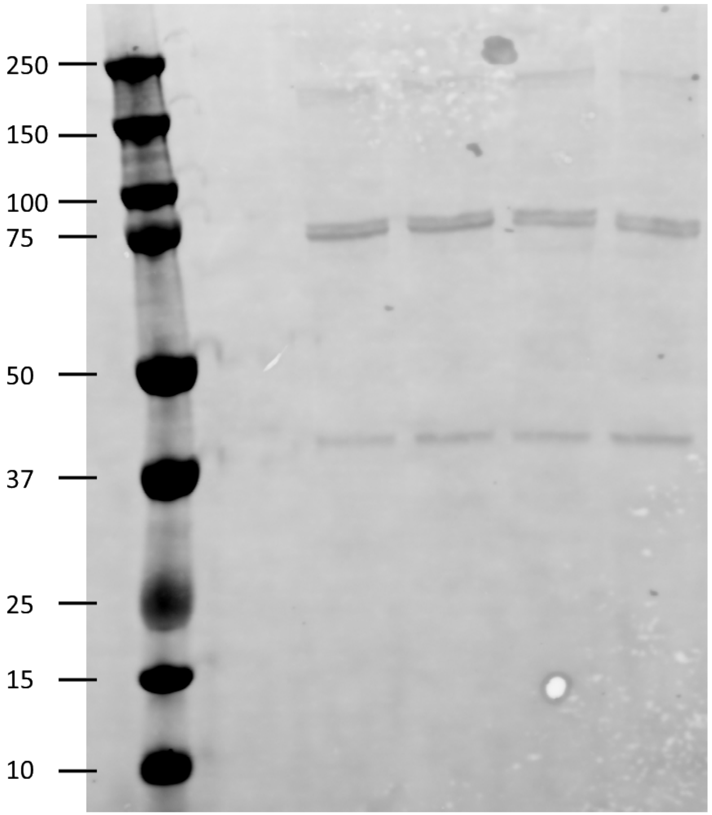

B

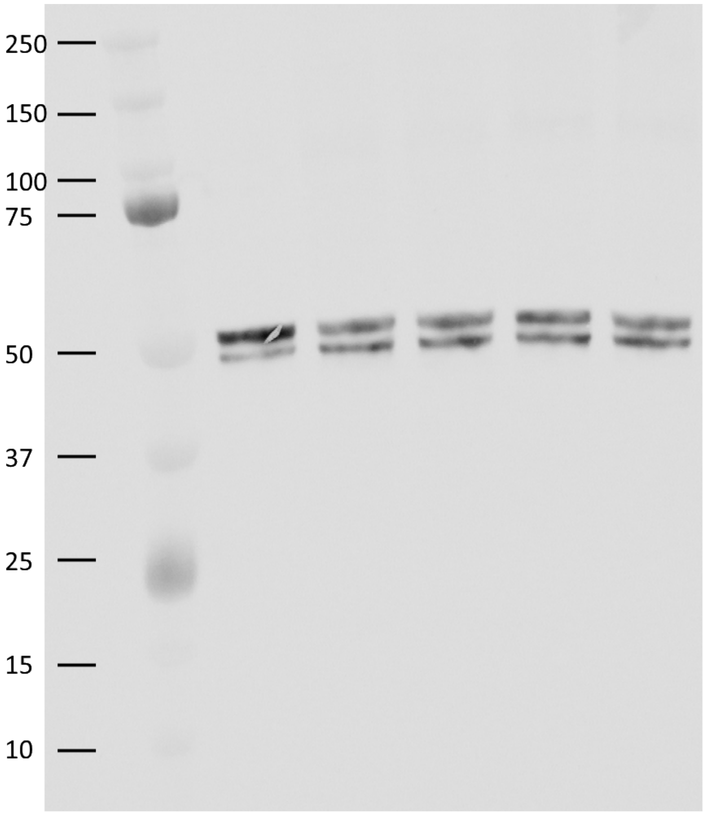

Supplement: Supplementary file 1 — Supplementary file1 (PDF 1486 KB) [file 18_2024_5149_MOESM1_ESM.pdf]
